# An update of neurological manifestations of vasculitides and connective tissue diseases: a literature review

**DOI:** 10.1590/S1679-45082015RW3308

**Published:** 2015

**Authors:** Anastasia Bougea, Evangelos Anagnostou, Nikolaos Spandideas, Nikolaos Triantafyllou, Evangelia Kararizou

**Affiliations:** 1 University of Athens Medical School, Athens, Greece.

**Keywords:** Central nervous system, Peripheral nervous system, Systemic vasculitis, Connective tissue diseases, Immunosuppressive agents/therapeutic use

## Abstract

Vasculitides comprise a heterogeneous group of autoimmune disorders, occurring as primary or secondary to a broad variety of systemic infectious, malignant or connective tissue diseases. The latter occur more often but their pathogenic mechanisms have not been fully established. Frequent and varied central and peripheral nervous system complications occur in vasculitides and connective tissue diseases. In many cases, the neurological disorders have an atypical clinical course or even an early onset, and the healthcare professionals should be aware of them. The purpose of this brief review was to give an update of the main neurological disorders of common vasculitis and connective tissue diseases, aiming at accurate diagnosis and management, with an emphasis on pathophysiologic mechanisms.

## INTRODUCTION

Traditionally, vasculitides are defined as a group of clinical and pathological entities characterized by an inflammatory cell infiltration and necrosis of blood vessel walls. They may be primary, involving large, medium and small vessels, or secondary, associated to infectious, malignant and connective tissue diseases (Chart 1). The latter cover a wide range group of multisystem inflammatory disorders involving muscles, joints, and skin, such as rheumatoid arthritis (RA), systemic lupus erythematosus (SLE), primary Sjögren’s syndrome (SS), and systemic sclerosis.^(1-4)^ Both immune-mediated changes and ischemia of the vascular wall – the hallmark of vasculitides, are the main causes of central nervous system (CNS) and peripheral nervous system (PNS) symptoms. However, due to complex spectrum of overlapping clinical manifestations, it is often difficult to recognize these disorders.

**Chart 1:** The most common classification of vasculitides adopted by International Chapel Hill Consensus Conference on the Nomenclature of Vasculitides(4)

Classification	Description
Large vessel vasculitis	Takayasu arteritis and temporal arteritis
Medium vessel vasculitis	Polyarteritis nodosa
Small vessel vasculitis	Antineutrophil cytoplasmic antibody-associated vasculitis, microscopic polyangiitis, granulomatosis with polyangiitis (Wegener's), eosinophilic granulomatosis with polyangiitis (Churg-Strauss)
Variable vessel vasculitis	Behçet disease and Cogan's syndrome
Single-organ vasculitis	Primary central nervous system vasculitis
Vasculitis associated with systemic disease	Lupus vasculitis
	Rheumatoid vasculitis
Vasculitis associated with probable etiology	Hydralazine-associated microscopic polyangiitis, hepatitis B virus–associated vasculitis, hepatitis C virus–associated vasculitis, cryoglobulinemic vasculitis

The neurological events are likely to be fatal without judicious use of immunosuppression, thus, prompt diagnosis can avoid pervasive injury and disability. Since much of the literature on vasculitis focuses on CNS involvement, very scarce data addressing both CNS and PNS manifestations have been published. This brief descriptive review aimed to update on recent information about primary and secondary vasculitides related to connective tissue diseases, and their most common neurological complications, diagnosis and management, with an emphasis on pathophysiologic mechanisms.

## METHODS

### Search strategy

A literature search was performed concerning neurological manifestations of vasculitides and connective tissue disorders using in the following databases: MEDLINE (from 1981 to 2014), LILACS (from 1983 to 2012), *Scientific Electronic Library Online* (SciELO) and Cochrane Library (from 1993 to 2012). The keywords used for the study were “peripheral nervous and central nervous complications”, “treatment” and “vasculitides and connective tissue disorders”. Articles, letters, summaries, dissertations and theses not published in Portuguese, English or Spanish were excluded, as well as studies that evaluated children or animal models. A particular emphasis was given to original articles and, on a secondary basis, to books and reviews, with special effort to identify the most recent publications.

The quality of studies was assessed using a Delphi list, with nine questions with three possible answers (yes, no and do not know), for internal and external validation, and statistical considerations.

## RESULTS

A total of 335 studies were identified, and 29 of them met the inclusion criteria established; they are summarized on chart 2.

**Chart 2:** Characterization of selected studies

Database	Title	Authors (year)	Type of study	Main themes
PubMed	Connective tissue disorders: systemic lupus erythematosus, Sjögren’s syndrome, and scleroderma	Streifler JY and Molad Y (2014)	Review	SLE, Sjögren’s syndrome, systemic sclerosis, (pathophysiology, diagnosis and treatment)
PubMed	Diagnosis and classification of central nervous system vasculitis	Hajj-Ali RA and Calabrese LH (2014)	Review	CNS vasculitis
PubMed	Cervical spine instability in rheumatoid arthritis	da Côrte FC and Neves N (2014)	Review	CNS involvement
PubMed	Update on the use of biologics in vasculitides	Vishwanath S, Relan M, Shen L and Ambrus JL Jr. (2014)	Review	Treatment
PubMed	Neuro-Behçet syndrome	Saip S, Akman-Demir G and Siva A (2014)	Review	CNS involvement, diagnosis and treatment
PubMed	Primary angiitis of the central nervous system and reversible cerebral vasoconstriction syndrome	Hammad TA and Hajj- Ali RA (2013)	Review	CNS involvement
PubMed	New-onset multiple sclerosis associated with adalimumab treatment in rheumatoid arthritis: a case report and literature review	Matsumoto T, Nakamura I, Miura A, Momoyama G and Ito K (2013)	Case report and literature review	CNS involvement
PubMed	Neurologic involvement in scleroderma: a systematic review	Amaral TN, Peres FA, Lapa AT, Marques-Neto JF and Appenzeller S (2013)	Systematic review	CNS + PNS involvement
PubMed	Characteristics of neurological manifestations of Behçet’s disease: a retrospective monocentric study in Tunisia	Houman MH, Bellakhal S, Ben Salem T, Hamzaoui A, Braham A, Lamloum M, et al. (2013)	Rectrospective	CNS involvement
PubMed	Neurological complications of Behçet’s syndrome	Kidd D (2012)	Review	CNS involvement and treatments
PubMed	Peripheral neuropathies in Sjögren’s syndrome: a critical update on clinical features and pathogenetic mechanisms	Pavlakis PP, Alexopoulos H, Kosmidis ML, Mamali I, Moutsopoulos HM, Tzioufas AG. et al. (2012)	Review	PNS involvement
PubMed	Addition of infliximab to standard therapy for ANCA-associated vasculitis	Morgan MD, Drayson MT, Savage CO and Harper L (2011)	Cohort	Treatment
PubMed	Infliximab or rituximab for refractory Wegener’s granulomatosis: long-term follow-up. A prospective randomised multicentre study on 17 patients	DeMenthon M, Cohen P, Pagnoux C, Buchler M, Sibilia J, Détrée F, et al. (2011)	Randomized	Treatment
PubMed	Churg-Strauss syndrome complicated by neuropathy: a clinicopathological study of nine cases	Kararizou E, Davaki P, Spengos K and Dimitracopoulos A (2011)	Clinical trial	CNS + PNS involvement
PubMed	Neurologicalinvolvement in Wegener’s granulomatosis	Holle JU and Gross WL (2011)	Systematic review	PNS +CNS involvement
PubMed	Clinical manifestations of neurological involvement in primary Sjögren’s syndrome	Gono T, Kawaguchi Y, Katsumata Y, Takagi K, Tochimoto A, Baba S, et al. (2011)	Cohort	PNS +CNS involvement
PubMed	Isolated cerebral vasculitis associated with rheumatoid arthritis	Caballol Pons N, Montalà N, Valverde J, Brell M, Ferrer I, Martinez-Yélamos S (2010)	Case	CNS involvement
PubMed	Neurologic complications of Churg-Strauss syndrome - a prospective monocentric study	Wolf J, Bergner R, Mutallib S, Buggle F and Grau AJ (2010)	Prospective	PNS involvement + treatment
PubMed	Treatment of polyarteritis nodosa and microscopic polyangiitis without poor-prognosis factors: A prospective randomized study of one hundred twenty-four patients	Ribi C, Cohen P, Pagnoux C, Mahr A, Arène JP, Puéchal X, et al. (2010)	Randomized	Treatment
PubMed	The neurologic manifestations of systemic lupus erythematosus	Greenberg BM (2009)	Review	Treatment
PubMed	The involvement of the peripheral nervous system in biopsy proven active giant cell arteritis	Pfadenhauer K, Roesler A and Golling A (2007)	Case series	PNS involvement
PubMed	Infliximab for maintenance of glucocorticosteroid induced remission of giant cell arteritis: a randomized trial	Hoffman GS, Cid MC, Rendt-Zagar KE, Merkel PA, Weyand CM, Stone JH, et al. (2007)	Randomized controlled trial	Treatment
PubMed	Nonsystemic vasculitic neuropathy: a clinicopathological study of 22 cases	Kararizou E, Davaki P, Karandreas N, Danou R, Vassilopoulos D (2005)	Clinical trial	PNS involvement
PubMed	Vasculitis of the nervous system	Younger DS (2004)	Review	PNS + CNS involvement
PubMed	Low dose MTX for progressive neuro-Behçet’s disease. A follow-up study for 4 years	Kikuchi H, Aramaki K and Hirohata S (2003)	Case series	Treatment
PubMed	Neuromuscular complications of connective tissue diseases	Rosenbaum R (2001)	Review	PNS involvement of RA, Sjögren’s syndrome, SLE, systemic sclerosis, vasculitis
PubMed	Diagnosis and management of isolated angiitis of the central nervous system	Moore PM (1989)	Review	Treatment
PubMed	Isolated benign cerebral vasculitis	Bettoni L, Juvarra G, Bortone E and Lenchi A (1984)	Case +review	CNS involvement

SLE: systemic lupus erythematosus; CNS: central nervous system; PNS: peripheral nervous system; RA: rheumatoid arthritis.

## DISCUSSION

To present the results, the articles were grouped according to the size of the vessels for each vasculitis, according to international classification.^(4)^ They were critically analyzed by pointing out the consensus as well as the yet unclear aspects related to mechanisms, diagnosis and treatment.

### Large vessel vasculitis

#### Temporal arteritis

Temporal arteritis, otherwise known as giant cell arteritis, affects the temporal artery of middle-aged or elderly patients. Notwithstanding its name, giant cells are not a requirement for diagnosis; histopathological findings are consistent with diffuse vascular involvement.

Clinical manifestations of the CNS are related to the anatomical region of the carotid artery. The most common and initial symptom is migraine (33%) localized in the temporal area, accompanied by fever, malaise, myalgia and anorexia. The temporal artery is often swollen and sensitive to palpation. The most common and serious complication of temporal arteritis is that of unilateral or bilateral loss of vision due to ischemic optic neuropathy. Blood tests reveal lymphocytosis and an increased erythrocyte sedimentation rate, while temporal artery biopsy confirms the presence of inflammation and giant cells.

PNS complications affect 15% of patients and include mononeuritis multiplex or distal symmetrical sensorimotor polyneuropathy.^(5)^

Polymyalgia rheumatica coexists in 40% of patients with temporal arteritis. Given the significant risk of vision loss, glucocorticoids should be started with no delay. A randomized controlled trial of 44 patients showed that maintenance therapy with infliximab failed to show more efficacy in disease control than that of steroids, and it did not allow a reduction in the dose of steroids required to prevent relapse.^(6)^ A dramatic response to low-dose corticosteroids remains a valuable tool in patients with uncertain diagnosis. However, the challenge lies in recognizing atypical cases that lack the more specific manifestations. We conclude that the diagnosis of giant cell arteritis should always be considered in elderly patients with an unexplained elevation of inflammatory markers and other neglected symptoms, such as trismus, facial edema and chronic dry cough, to avoid severe complications.

#### Takayasu disease

Takayasu disease is a granulomatous vasculitis causing stenosis and aneurysmal dilatation of large arteries, such as the aorta and its major branches in patients <40 years.^(7)^ Neurological dysfunction may be the initial manifestation, but more often it occurs later in the disease course. Ischemic optic neuropathy, other isolated cranial nerve palsies and stroke, have been reported due to involvement of the internal carotid artery or its branches. Compared to the CNS, the involvement of the PNS in form of vasculitic neuropathy is much rarer, with subacute sensorimotor deficit in a cervicobrachial plexus distribution.^(7) ^Despite the fact that no prospective controlled study has been conducted to date, anti-tumor necrosis factor alpha could be a good therapeutic option in takayasu arthritis, who were unable to achieve or maintain remission with steroids alone or cyclophosphamide, or low-dose methotrexate. Recently, in rheumatic patients on TNF-α blocking agents, no response to this treatment was noted, which suggests the presence of different pathogenic mechanism.^(8)^

## Medium vessel vasculitis

### Polyarteritis nodosa

Neurologic manifestations are very common in polyarteritis nodosa (PAN) due to systemic necrotizing vasculitis that affects medium-sized arteries and cause ischemia, leading to thrombosis or bleeding.^(1-4,9)^ CNS symptoms occur relatively late in the course of the disease, causing multifocal encephalopathy in 40% of patients, depending on the region in which lesions appear. Patients with PAN typically demonstrate CNS neurological signs, such as personality and memory disorders, atypical persistent headaches, aphasia, hemiplegia, visual disturbance (blurred vision and hemianopia), seizures, transverse myelitis and subarachnoid hemorrhage. Up to 65% of patients present with PNS disorders, including mononeuritis multiplex (almost typical and generally painful manifestation of the disease) and distal symmetric sensorimotor polyneuropathy.^(2,10)^ PAN patients with no poor prognostic factors upon diagnosis and treated initially with corticosteroids alone usually present an excellent survival in the long run.^(8)^ However, relapses are frequent in severe cases, with neurologic, renal or cardiac manifestations. Cyclophosphamide plus corticosteroids could change to a better outcome.^(10,11)^

## Small vessel vasculitis

### Allergic granulomatosis (Churg-Strauss syndrome)

Churg-Strauss syndrome is a rare form of systemic vasculitis occurring in patients with asthma and eosinophilia, according to the 1990 criteria, from the American College of Rheumatology.^(12)^ The neurological findings in allergic granulomatosis are caused by systemic necrotizing vasculitis, with eosinophil infiltrate affecting small vessels. CNS events are rare and resemble nodular polyarteritis.^(1,9,11,13)^ CNS involvement may include paralysis of seventh cranial nerve and phrenic nerve palsy, cerebral hemorrhage or infarction, seizures and coma, but these events are much less typical.

On the other hand, PNS involvement is noted in about 60% of patients, mainly in the form of mononeuritis multiplex or symmetric mild sensory axonal neuropathy.^(2,3,11-14)^ The neuropathy is caused mainly by nerve ischemia due to occlusion of *vasa nervorum*. The result of this infarction is a loss of sensory and motor axons. Nerve biopsy is characteristic and useful to confirm diagnosis. Churg and Strauss reported three primary histopathological alterations: eosinophilic tissue infiltration, necrotizing vasculitis and extravascular granulomas. The inflammatory process in Churg-Strauss syndrome tends to affect smaller epineural arterioles smaller than those in systemic vasculitis.^(12)^

It can be conclude that Churg-Strauss syndrome frequently presents polyneuropathy as a complication; and since its remission depends on immunosuppression therapy, it is important to recognize it at an early stage. The diagnosis of polyneuropathy is based on clinical and electrophysiologic studies, but precise histology, immunohistochemistry and morphometric studies of the peripheral nerve biopsy may be decisive to make diagnosis.

### Polyangeitis granulomatosa

Approximately 50% of patients with polyangeitis granulomatosa present with neurological complications caused by necrotizing granulomatous lesions of small vessels.^(1-4,9)^ CNS involvement is diverse, depending on the presence of vasculitis, contiguous extension, or remote granulomatous spread. Granulomas cause basilar meningitis, temporal lobe dysfunction and venous sinus occlusion.^(9)^ Cranial neuropathy of nerves II, VI and VII are also caused by granulomatous lesions.^(14)^

The most typical and frequent PNS complication is mononeuritis multiplex (10 to 22%). Symmetrical sensorimotor polyneuropathy with relatively rapid progression, though rare, was also reported.^(15)^ Based on the 1994 Chapel Hill Consensus, the diagnosis of polyangeitis granulomatosa requires a tissue biopsy showing evidence of vasculitis, demonstrated by granulomatous inflammation and necrosis in the involved organs.^(16)^ As to CNS and PNS involvement, rituximab and infliximab emerged as potential treatment options for refractory disease.^(17)^ Thirty-three patients with active disease were enrolled in an open prospective trial that reviewed adding infliximab to standard therapy, aiming to achieve remission within a mean follow-up period of 12 months.^(18)^ No benefit was demonstrated with the use of anti-tumor necrosis factor alpha agents. Current treatment strategies have substantial short-term and long-term adverse effects, and relapses are frequent; thus, less-toxic and more-effective approaches are needed.

## Variable vessel vasculitis

### Behçet syndrome

This multisystem disorder of unknown etiology predominantly affects men with oral and genital ulceration and uveitis, based on the International Criteria for Behçet Disease.^(19)^ The histology reveals vasculitis of small vessels and perivascular deposition of inflammatory cells in the meninges.

CNS involvement in Behçet disease, usually called neuro-Behçet syndrome (NB), occur in 30% of cases and includes acute and chronic progressive forms. Acute NB is characterized by acute meningoencephalitis with focal lesions, presenting high intensity areas in T2-weightened images or FLAIR images on magnetic resonance imaging.^(20)^ Cyclosporin A frequently causes acute NB. Acute NB responds to steroid therapy, and is usually self-limiting. By contrast, chronic progressive NB is characterized by intractable slowly progressive dementia, ataxia and dysarthria, with persistent elevation of cerebrospinal fluid (CSF) interleukin 6 (IL-6) activity (more than 20pg/mL). PNS involvement is extremely rare, although isolated cases of distal symmetrical polyneuropathy and mononeuritis multiplex have been reported.^(21)^

Chronic progressive NB is resistant to conventional treatment with steroids, cyclophosphamide or azathioprine. Recent studies have reported on the efficacy of low dose methotrexate in chronic progressive NB.^(22)^ Nevertheless, symptomatic management is limited to intravenous high-dose methylprednisolone, followed by a prolonged oral taper. On the other hand, preventive treatment includes azathioprine, cyclophosphamide, interferon alpha and anti-tumor necrosis factor alpha agents for long-term, although there is no evidence in multicenter studies about their efficacy.^(23)^

## Single-organ vasculitis

### Primary angiitis of the central nervous system

Primary angiitis of the CNS (PACNS) is a rare inflammatory disorder that may involve both small and medium-sized leptomeningeal, cortical and subcortical arteries of the CNS. The histologic findings of PACNS comprise granulomatous inflammation, fibrinoid necrosis of vessel walls or exclusively lymphocytic cell infiltrates.^(24-26)^ Subacute or chronic meningeal type form is observed, mainly in male individuals, and present with headache, or migraine lasting 3 to 6 months, followed by focal and generalized neurologic symptoms. Confusion, impaired memory and concentration are signs of cognitive dysfunction. Others CNS symptoms include hemiparesis, seizures, ataxia and cranial nerve palsy. The CSF examination shows a slight increase in cells and albumin. However, the clinical findings and brain imaging examinations are relatively nonspecific, making diagnosis even more difficult. Brain biopsy reveals the characteristic histological lesions of the disease.^(27)^ Reversible cerebral vasoconstriction syndrome is a main condition that mimics PACNS, with recurrent thunderclap headache, with or without neurological deficit, and normal CSF analysis findings. Magnetic resonance angiography shows reversible diffuse segmental vasospasm of intracranial vessels.^(28)^ Since no controlled study on CNS angiitis treatment has been performed, the recommendations for PACNS management are based on protocols for systemic vasculitides with severe organ involvement. A combination of steroids and pulse cyclophosphamide is recommended for patients with a poor prognosis.^(24,25)^ With a relapse rate of 25% and reduced survival rate, a close follow-up of suspected PACNS is mandatory.

## Vasculitides secondary to connective tissue diseases

### Rheumatoid arthritis

RA is the most common disease of the connective tissue and it mainly affects the joints. Nervous system symptoms are primarily due to vasculitis and damage resulting from the pressure applied by rheumatoid nodules.^(1,5,29)^ The incidence of rheumatoid vasculitis has diminished substantially lately. CNS involvement is uncommon in RA and presents vasculitis-like symptoms, such as stroke, seizures, and meningitis (probably due to rheumatoid nodules).

Recent theories on the pathogenesis of RA suggest that the synovial cells of these patients chronically express an antigen that triggers the production of the rheumatoid factor involving the polymorphonuclear leukocyte infiltrate. Destructive synovitis results in ligament laxity and bone erosion, and atlantoaxial subluxation is the most common cervical deformity associated with RA. Since neurologic deficits are observed in only 7 to 34% of cases, many patients with pain and radiographic criteria for instability do not develop neurologic sequelae. However, 10% of patients die due to brainstem compression.^(30)^

PNS complications are very common and depend on the cause, site and severity of the injury, and include: (1) sensory peripheral neuropathy, attributed to vasculitis characterizing the disease, though the pathogenesis remains unknown. According to clinical and electrophysiological findings, aesthetic impairment is recorded in over 75% of patients. Neuropathy is predominantly axial and manifests with mild sensory symptoms in the limbs, characterized by a symmetric and progressive disease course;^(2,3) ^(2) mononeuritis multiplex is less frequent in RA but has a more severe clinical course;^(2)^ (3) peripheral entrapment neuropathy is caused by direct pressure on a single nerve. The nerves most commonly affected are the median nerve (carpal tunnel syndrome); the ulnar nerve in the elbow; Guyon canal at the wrist; medial or lateral plantar nerve in the tarsal canal; and the peroneal nerve;^(2,3)^ (4) sensorimotor peripheral neuropathy in the upper and lower limbs is more severe and has a poor prognosis.^(2,3)^

Muscle weakness and atrophy, especially in proximal sites, are frequent in patients with RA. Muscle biopsy reveals evidence of myositis in about 40% of patients, while levels of muscle enzymes are always elevated.^(29)^

The treatment of RA can cause neurological side effects. Gold is the cause of Guillain-Barré in 1% of patients characterized by sudden onset and rapidly progressive muscle weakness. Albeit rare, cranial nerve palsies, transverse myelitis and seizures were also reported. Chloroquine may cause headaches, psychotic disorders, neuropathy and myopathy, while D- penicillamine is often responsible for taste changes, inflammatory myopathy or myasthenia. Hearing disorders, particularly at high frequencies are associated with high doses of salicylates, as well as with the known side effects of corticosteroids. Demyelination observed in RA patients receiving anti-tumor necrosis factor alpha treatment could be attributed to the unmasking of latent preexisting multiple sclerosis (MS), the emergence of a new demyelinating event (either MS or MS-like), or to the incidental coexistence of the two disorders.^(31)^ Nevertheless, morbidity and mortality rates remain high, despite aggressive treatment with cyclophosphamide or biologic agents.

### Systemic lupus erythematosus

SLE is an autoimmune disease characterized by multisystem organ involvement, heterogeneity of clinical features, and varied severity levels, and similarities with other autoimmune diseases. Thus, the diseases that mimic lupus may present as a lupus-like condition (*i.e*., two or three criteria) or as a disease that meets the classification criteria for SLE.^(32)^ Multiple sites may be involved in the nervous system, often in the early stages, especially among young people.^(1,4,9,33)^

CNS manifestations range from 33 to 75% among patients with SLE, mimicking other neurological conditions.^(33,34)^

*– *Non-thrombotic disease of the CNS in which antibodies against structural components of the CNS, immune complexes, vasculitis and non-vascular lesions are considered mainly responsible. It is often characterized by psychoses, seizures, headaches (24 to 72%), cognitive dysfunction and chorea.

*– *Thrombotic disease of the CNS is probably due to multifactorial processes, such as accelerated atherosclerosis and prothrombotic state due to antiphospholipid antibodies (aPL).^(34)^ SLE patients with stroke or recurrent transient ischemic attacks should be evaluated for a valvular source of emboli (Libman-Sacks endocarditis), and for aPL, because anticoagulation is required in these patients.

The complications of the PNS in SLE are rare, and arise in approximately 10% of patients, due to vasculitis. Trigeminal neuropathy may precede other symptoms of the disease by several years, with distal symmetric sensory or sensorimotor polyneuropathy being the most commonly observed complication. It may be subacute or chronic, with mild to moderate symptoms. Few cases of acute or chronic inflammatory demyelinating polyradiculoneuropathy were reported.^(35)^ Generally, myopathies in SLE take the form of inflammatory myopathies when appearing in the acute phase of the disease or as secondary side effects, such as hypokalemia resulting from treatment with corticosteroids or hydroxychloroquine.^(33)^ Due to the lack of controlled randomized trials, current therapeutic approach is still empirical and based on clinical experience. The treatments with intravenous immunoglobulins, mycophenolate mofetil, rituximab, intratecal dexamethasone or methotrexate require further studies to confirm their usefulness.^(34)^

### Systemic sclerosis

Systemic sclerosis is characterized by widespread microvasculopathy and diffuse tissue fibrosis affecting the skin and other systemic organs, particularly heart, lungs, and gastrointestinal tract. Previously considered a rare event, neurological complications in systemic sclerosis have been increasingly recognized.^(1,5)^ In a recent review of 180 studies, CNS involvement in systemic sclerosis was characterized by headache (23.73%), seizures (13.56%) and cognitive impairment (8.47%). Depression and anxiety were frequently observed (73.15% and 23.95%, respectively).^(36)^ The most common peripheral neuropathy recorded in patients with systemic sclerosis is carpal tunnel syndrome (1 to 10%).^(2,3,34,36) ^Trigeminal neuropathy occurred most frequently in young women with systemic sclerosis, overlapping with other disorders, particularly mixed connective tissue disease, with clinical evidence of myositis.^(36)^ Some patients experience proximal muscle weakness and present with increased muscle enzymes, but few show evidence of inflammatory myopathy on muscle biopsy. Corticosteroids and cyclophosphamide may be effective.^(34,36)^

## Sjögren’s syndrome

SS is characterized by T-cell (CD4+) infiltration and destruction of salivary and lacrimal glands leading to loss of tears (keratoconjunctivitis sicca) and saliva (xerostomia). Neurological manifestations may precede the sicca symptoms in 40 to 93% of the cases. CNS complications observed in 15% of patients include trigeminal neuralgia, stroke, hemorrhage, seizures, aseptic meningoencephalitis, and transverse myelitis;^(34)^ however, the spectrum of neurological complication is not well defined. The vascular injury may be related to the presence of antineuronal antibodies and anti-Ro antibodies.^(4)^ Additionally, SS with CNS disease may mimic MS, suggesting other mechanisms rather than vasculopathy.^(1,4,37) ^The PNS is the most commonly affected in 30% of patients, mainly in women suffering from SS with peripheral symmetrical sensorimotor polyneuropathy and mononeuritis multiplex.^(2,3,38)^ Proinflammatory cytokines, such as tumor necrosis factor alpha, have been implicated, and some clinical improvement has seen with intravenous immunoglobulin therapy and anti-tumor necrosis factor alpha. It was recently observed that antibodies against the type-3 muscarinic receptor may eventually explain part of the broader autonomic dysfunction found in patients with SS.^(39)^ In conclusion, although CNS and PNS complications of SS are difficult to assess, partly because of the wide spectrum of possible manifestations, their incidence is estimated to be approximately 20%. Mononeuropathy remains the most specific complication.

## CONCLUSION

The vasculitides and connective tissue diseases associated to them provide an avenue for investigating the pathophysiology of immune system among the vasculature of the central and peripheral nervous system. The reported prevalence of central nervous system involvement varies widely; however, it is generally regarded to be less frequently involved than the peripheral nervous system. A pure sensory neuropathy is the most frequently peripheral nervous system manifestation; a long-term, insidious course is typically observed. The neurological manifestations are mainly caused by direct effect of the diseases, as well as side effects of immunotherapies. Searching the international bibliography, there is no established diagnostic tool neither effective treatment for these disorders. However, some limitations should be considered in this review, such as methods of reporting complications varied considerably, reported frequencies were determined from studies of varying sample sizes across multiple age groups. Prospective clinical trials are required for early recognition of all possible medication-related side effects. Long-term evaluation of patients is important in order to manage relapses.

### Take-home message

In this review we updated the new pieces of information for neurological complications, their diagnosis and management that physicians should be aware of.
