# Supplementary material for: An update of neurological manifestations of vasculitides and connective tissue diseases: a literature review
Source: Einstein (Sao Paulo). 2015 Oct-Dec;13(4):627–35. doi: 10.1590/S1679-45082015RW3308 (PMC4878643; doi:10.1590/S1679-45082015RW3308)
Supplement: Translation

# Translation: Atualização das manifestações neurológicas das vasculitides e das doenças do tecido conjuntivo: revisão de literatura

**Authors:** Anastasia Bougea, Evangelos Anagnostou, Nikolaos Spandideas, Nikolaos Triantafyllou, Evangelia Kararizou

**Affiliations:** 1 University of Athens Medical School, Atenas, Grécia.

**Keywords:** Sistema nervoso central, Sistema nervoso periférico, Vasculite sistêmica, Doenças do tecido conjuntivo, Imunossupressores/uso terapêutico

## Abstract

As vasculites são um grupo heterogêneo de doenças autoimunes primárias ou secundárias a uma grande variedade de doenças infecciosas sistêmicas, malignas ou do tecido conjuntivo. Estas últimas são as que ocorrem com mais frequência, porém os mecanismos patogênicos ainda não foram plenamente determinados. Diversas e frequentes complicações do sistema nervoso central e periférico ocorrem nas vasculites e doenças do tecido conjuntivo. Em muitos casos, os distúrbios neurológicos têm evolução clínica atípica ou mesmo início precoce, ao que todos os profissionais de saúde devem estar cientes. O objetivo desta breve revisão foi atualizar os principais distúrbios neurológicos da vasculite comum e das doenças do tecido conjuntivo, visando ao diagnóstico e ao tratamento corretos, com ênfase nos mecanismos fisiopatológicos.

## INTRODUÇÃO

Tradicionalmente, definem-se vasculites como um grupo de entidades clínico-patológicas caracterizadas por infiltrado de células inflamatórias e necrose da parede vascular. Podem ser primárias, envolvendo vasos de tamanho grande, médio e pequeno, ou secundárias, associadas a doenças infecciosas, malignas e do tecido conjuntivo (Quadro 1). Essas últimas cobrem amplo grupo de distúrbios inflamatórios multissistêmicos musculares, articulares e dermatológicos, como artrite reumatoide (AR), lúpus eritematoso sistêmico (LES), síndrome de Sjögren (SS) primária, e esclerose sistêmica.^(1-4)^ As alterações imunomediadas e a isquemia da parede vascular, a marca registrada das vasculites, são as principais causas dos sintomas dos sistemas nervosos central (SNC) e sistema nervoso periférico (SNP). No entanto, devido à sobreposição de um complexo espectro de manifestações clínicas, o reconhecimento desses distúrbios geralmente é difícil.

**Quadro 1:** Classificação mais comum de *vasculitis*, adotada na *International Chapel Hill Consensus Conference Nomenclature of Vasculitides*.(4)

Classificação	Descrição
Vasculite de grandes vasos	Arterite de Takayasu e arterite temporal
Vasculite de vasos médios	Poliarterite nodosa
Vasculite de vasos pequenos	Vasculite associada a anticorpo anticitoplasma de neutrófilos, poliangeíte microscópica, granulomatose com poliangeíte (de Wegener), granulomatose eosinofílica com poliangeíte (Churg-Strauss)
Vasculite de vasos de calibre variável	Síndrome de Behçet e síndrome de Cogan
Vasculite de órgão único	Angeíte primária do sistema nervoso central
Vasculite associada à doença sistêmica	Vasculite lúpica
	Vasculite reumatoide
Vasculite associada à etiologia provável	Poliangeíte microscópica associada à hidralazina, vasculite associada ao vírus da hepatite B, vasculite associada ao vírus da hepatite C, vasculite crioglobulinêmica

Os eventos neurológicos tendem a ser fatais sem o uso criterioso de terapias imunossupressoras, de modo que um rápido diagnóstico pode evitar lesões disseminadas e deficiências. Como grande parte da literatura sobre vasculites concentra-se no envolvimento do SNC, há bem poucos dados publicados que abordam tanto as manifestações no SNC quanto no SNP. Esta breve revisão descritiva visa atualizar as recentes informações sobre vasculites primárias e secundárias das doenças do tecido conjuntivo com suas complicações neurológicas mais comuns, seu diagnóstico e tratamento, dando ênfase aos mecanismos fisiopatológicos.

## MÉTODOS

### Estratégia de busca

Foi realizada uma busca na literatura no tocante a manifestações neurológicas das vasculites e doenças do tecido conjuntivo usando os seguintes bancos de dados: MEDLINE (de 1981 a 2014); LILACS (de 1983 a 2012), *Scientific Electronic Library Online* (SciELO) e *Cochrane Library* (de 1993 a 2012). Os descritores usados para o estudo foram “complicações do sistema nervoso periférico”, “complicações do sistema nervoso central”, “tratamento” e “vasculites e doenças do tecido conjuntivo”. Foram excluídos artigos, cartas, resumos, dissertações em outros idiomas, além do português, inglês ou espanhol, bem como estudos que avaliavam crianças ou modelos animais. Deu-se ênfase especial aos artigos originais e, em segundo lugar, a livros e revisões, com especial empenho na identificação dos mais recentes.

A qualidade dos estudos foi avaliada usando-se uma lista *Delphi* contendo nove perguntas, com três possíveis respostas (sim, não e não sei), para validação interna e externa, e considerações estatísticas.

## RESULTADOS

Foram identificados 335 estudos, dos quais 29 satisfizeram os critérios de inclusão estabelecidos, que estão resumidos no quadro 2.

**Quadro 2:** Caracterização dos estudos selecionados

Banco de dados	Título	Autores (ano)	Tipo de estudo	Principais temas
PubMed	*Connective tissue disorders: systemic lupus erythematosus, Sjögren’s syndrome, and scleroderma*	Streifler JY e Molad Y (2014)	Revisão	LES, síndrome de Sjögren, esclerose sistêmica, (fisiopatologia, diagnóstico e tratamento)
PubMed	*Diagnosis andclassificationof central nervous systemvasculitis*	Hajj-Ali RA e Calabrese LH (2014)	Revisão	Vasculite no SNC
PubMed	*Cervical spine instability in rheumatoid arthritis*	da Côrte FC e Neves N (2014)	Revisão	Comprometimento do SNC
PubMed	*Update on the use of biologics in vasculitides*	Vishwanath S, Relan M, Shen L e Ambrus JL Jr. (2014)	Revisão	Tratamento
PubMed	*Neuro-Behçet syndrome*	Saip S, Akman-Demir G e Siva A (2014)	Revisão	Comprometimento do CNS, diagnóstico e tratamento
PubMed	*Primary angiitis of the central nervous system and reversible cerebral vasoconstriction syndrome*	Hammad TA e Hajj- Ali RA (2013)	Revisão	Comprometimento do SNC
PubMed	*New-onset multiple sclerosis associated with adalimumab treatment in rheumatoid arthritis: a case report and literature review*	Matsumoto T, Nakamura I, Miura A, Momoyama G e Ito K (2013)	Relato de caso e revisão da literatura	Comprometimento do SNC
PubMed	*Neurologic involvement inscleroderma: a systematic review*	Amaral TN, Peres FA, Lapa AT, Marques-Neto JF e Appenzeller S (2013)	Revisão sistemática	Comprometimento do SNC e SNP
PubMed	*Characteristics ofneurologicalmanifestations of Behçet’s disease: a retrospective monocentric study in Tunisia*	Houman MH, Bellakhal S, Ben Salem T, Hamzaoui A, Braham A, Lamloum M, et al. (2013)	Retrospectivo	Comprometimento do SNC
PubMed	*Neurologicalcomplicationsof Behçet’ssyndrome*	Kidd D (2012)	Revisão	Comprometimento do SNC e tratamentos
PubMed	*Peripheral neuropathies in Sjögren’s syndrome: a critical update on clinical features and pathogenetic mechanisms*	Pavlakis PP, Alexopoulos H, Kosmidis ML, Mamali I, Moutsopoulos HM, Tzioufas AG, et al. (2012)	Revisão	Comprometimento do SNP
PubMed	*Addition of infliximab to standard therapy for ANCA-associated vasculitis*	Morgan MD, Drayson MT, Savage CO e Harper L (2011)	Coorte	Tratamento
PubMed	*Infliximab or rituximab for refractory Wegener’s granulomatosis: long-term follow-up. A prospective randomised multicentre study on 17 patients*	DeMenthon M, Cohen P, Pagnoux C, Buchler M, Sibilia J, Détrée F, et al. (2011)	Randomizado	Tratamento
PubMed	*Churg-Strauss syndrome complicated by neuropathy: a clinicopathological study of nine cases*	Kararizou E, Davaki P, Spengos K e Dimitracopoulos A (2011)	Ensaio clínico	Comprometimento do SNC e SNP
PubMed	*Neurologicalinvolvement in Wegener’s granulomatosis*	Holle JU e Gross WL (2011)	Revisão sistemática	Comprometimento do SNC e SNP
PubMed	*Clinical manifestations of neurological involvement in primary Sjögren’s syndrome*	Gono T, Kawaguchi Y, Katsumata Y, Takagi K, Tochimoto A, Baba S, et al. (2011)	Coorte	Comprometimento do SNC e SNP
PubMed	*Isolated cerebral vasculitis associated with rheumatoid arthritis*	Caballol Pons N, Montalà N, Valverde J, Brell M, Ferrer I, Martinez-Yélamos S (2010)	Caso	Comprometimento do SNC
PubMed	*Neurologic complications of Churg-Strauss syndrome - a prospective monocentric study*	Wolf J, Bergner R, Mutallib S, Buggle F e Grau AJ (2010)	Prospectivo	Comprometimento do SNP e tratamento
PubMed	*Treatment of polyarteritis nodosa and microscopic polyangiitis without poor-prognosis factors: A prospective randomized study of one hundred twenty-four patients*	Ribi C, Cohen P, Pagnoux C, Mahr A, Arène JP, Puéchal X, et al. (2010)	Randomizado	Tratamento
PubMed	*The neurologic manifestations of systemic lupus erythematosus*	Greenberg BM (2009)	Revisão	Tratamento
PubMed	*The involvement of the peripheral nervous system in biopsy proven active giant cell arteritis*	Pfadenhauer K, Roesler A e Golling A (2007)	Série de casos	Comprometimento do SNP
PubMed	*Infliximab for maintenance of glucocorticosteroid induced remission of giant cell arteritis: a randomized trial*	Hoffman GS, Cid MC, Rendt-Zagar KE, Merkel PA, Weyand CM, Stone JH, et al. (2007)	Randomizado	Tratamento
PubMed	*Nonsystemic vasculitic neuropathy: a clinicopathological study of 22 cases*	Kararizou E, Davaki P, Karandreas N, Danou R, Vassilopoulos D (2005)	Ensaio clínico	Comprometimento do SNP
PubMed	*Vasculitis of the nervous system*	Younger DS (2004)	Revisão	Comprometimento do SNP + SNC
PubMed	*Low dose MTX for progressive neuro-Behçet’s disease. A follow-up study for 4 years*	Kikuchi H, Aramaki K e Hirohata S (2003)	Série de casos	Tratamento
PubMed	*Neuromuscular complications of connective tissue diseases*	Rosenbaum R (2001)	Revisão	Comprometimento do SNP na AR, síndrome de Sjögren, LES, esclerose sistêmica e vasculite
PubMed	*Diagnosis and management of isolated angiitis of the central nervous system*	Moore PM (1989)	Revisão	Tratamento
PubMed	*Isolated benign cerebral vasculitis*	Bettoni L, Juvarra G, Bortone E e Lenchi A (1984)	Caso + revisão	Comprometimento do SNC

LES: lúpus eritematoso sistêmico; SNC: sistema nervoso central; SNP: sistema nervoso periférico; AR: artrite reumatoide.

## DISCUSSÃO

Na apresentação dos resultados, os artigos foram agrupados pelo tamanho dos vasos para cada vasculite, de acordo com a classificação internacional.^(4)^ Eles foram analisados criticamente, indicando-se o consenso e os aspectos ainda obscuros referentes a mecanismos, diagnóstico e tratamento.

### Vasculite de grandes vasos

#### Arterite temporal

A arterite temporal, também conhecida como arterite de células gigantes, afeta a artéria temporal de pacientes de meia-idade ou idosos. A despeito do nome, as células gigantes não são requisito para o diagnóstico. Os achados histopatológicos são condizentes com comprometimento vascular difuso.

As manifestações clínicas do SNC estão relacionadas à região anatômica da artéria carótida. O sintoma inicial e mais comum é a enxaqueca (33%) localizada na região temporal, acompanhada por febre, mal-estar, mialgia e anorexia. A artéria temporal se encontra geralmente ingurgitada e sensível à palpação. A complicação mais comum e grave da arterite temporal é a perda unilateral ou bilateral da visão, devido à neuropatia isquêmica do nervo óptico. Os exames de sangue revelam linfocitose e elevação da velocidade de hemossedimentação, ao passo que a biópsia da artéria temporal confirma a presença de inflamação e células gigantes.

As complicações do SNP afetam 15% dos pacientes e incluem polineuropatia sensitivo-motora distal simétrica ou mononeuropatia múltipla.^(5)^

A polimialgia reumática coexiste em 40% dos pacientes com arterite temporal. Devido ao risco significativo de perda da visão, o tratamento com glicocorticoides deve ser iniciado sem demora. Estudo controlado randomizado de 44 pacientes mostrou que a terapia de manutenção com infliximabe não apresentou mais eficácia no controle da doença do que a terapia com esteroides, e tampouco permitiu uma redução na dose necessária de corticoides para prevenir recaídas.^(6)^ A resposta dramática ao tratamento com baixa dose de corticosteroides continua a ser uma valiosa ferramenta nos pacientes com diagnóstico incerto. No entanto, o desafio está no reconhecimento de casos atípicos, que carecem de manifestações mais específicas. Conclui-se que o diagnóstico de arterite de células gigantes sempre deve ser levado em consideração em pacientes idosos com elevação inexplicável de marcadores inflamatórios e outros sintomas negligenciados, como trismo, edema facial ou tosse seca crônica, a fim de evitar complicações graves.

#### Doença de Takayasu

A doença de Takayasu é uma vasculite granulomatosa que causa estenose e dilatação aneurismática das grandes artérias, como a aorta e seus principais ramos, em pacientes com idade <40 anos.^(7)^ A disfunção neurológica pode ser a manifestação inicial, porém ela ocorre com mais frequência posteriormente no curso da doença. Foram relatadas neuropatia óptica isquêmica, outras paralisias de nervos cranianos isolados e acidente vascular cerebral (AVC), devido ao envolvimento da artéria carótida interna ou de seus ramos. Comparado ao SNC, o envolvimento do SNP na forma de neuropatia vasculítica é bem mais raro, com défice sensitivo-motor subagudo em uma distribuição do plexo cervicobraquial.^(7)^ Apesar de nenhum estudo prospectivo controlado ter sido realizado até o momento, o tratamento com antifator de necrose tumoral alfa pode ser uma boa opção terapêutica em pacientes com doença de Takayasu, em que não foi possível obter ou manter a remissão com uso exclusivo de esteroides ou ciclofosfamida, ou com metotrexato em doses baixas. Recentemente, em paciente reumáticos em tratamento com agentes bloqueadores de fator de necrose tumoral alfa, nenhuma resposta foi notado, o que sugere a presença de um mecanismo patogenético diferente.^(8)^

## Vasculite de vasos médios

### Poliarterite nodosa

As manifestações neurológicas são muito comuns na poliarterite nodosa (PAN), devido à vasculite necrosante sistêmica, que afeta artérias médias e causa isquemia, o que leva à trombose ou ao sangramento.^(1-4,9)^ Os sintomas do SNC ocorrem relativamente tarde no curso da doença e causam encefalopatia multifocal em 40% dos pacientes, dependendo da região em que aparecerem as lesões. Os pacientes com PAN geralmente apresentam sinais neurológicos do SNC, como distúrbios de personalidade e de memória, cefaleia atípica persistente, afasia, hemiplegia, perturbações visuais (visão borrada e hemianopsia), convulsões, mielite transversa e hemorragia subaracnoide. Até 65% dos pacientes apresentam distúrbios do SNP, que incluem mononeurite múltipla (manifestação quase típica e geralmente dolorosa da doença) e polineuropatia sensitivo-motora distal simétrica.^(2,10)^ Os pacientes com PAN, que não apresentam fatores de mau prognóstico quando diagnosticados e que são tratados inicialmente apenas com corticosteroides, têm, em geral, excelente sobrevida a longo prazo.^(8)^ Contudo, as recidivas são frequentes nos casos graves, com manifestações neurológicas, renais ou cardíacas. Um tratamento com ciclofosfamida e corticosteroides pode melhorar o desfecho da doença.^(10,11)^

## Vasculite de pequenos vasos

### Granulomatose alérgica (síndrome de Churg-Strauss)

A síndrome de Churg-Strauss é uma forma rara de vasculite sistêmica que ocorre em pacientes com asma e eosinofilia, de acordo com os critérios do *American College of Rheumatology*, de 1990.^(12)^ Os achados neurológicos na granulomatose alérgica são causados por vasculite necrosante sistêmica, com infiltrado de eosinófilos que afeta vasos de pequeno calibre. Os eventos do SNC são raros e se assemelham à poliarterite nodular.^(1,9,11,13)^ O envolvimento do SNC pode incluir paralisia do sétimo nervo craniano e paralisia do nervo frênico, hemorragia ou infarto cerebral, convulsões e coma, mas esses eventos são bem menos comuns.

Por outro lado, o envolvimento do SNP é observado em cerca de 60% dos pacientes, principalmente sob a forma de mononeurite múltipla ou neuropatia axonal sensitiva simétrica leve.^(2,3,11-14)^ A neuropatia é causada principalmente por isquemia do nervo devido à oclusão de *vasa nervorum*. O resultado desse infarto é uma perda de axônios sensitivos e motores. A biópsia de nervo é característica e útil para confirmar o diagnóstico. Churg e Strauss relataram três alterações histopatológicas primárias: infiltrado eosinofílico tecidual, vasculite necrosante e granulomas extravasculares. O processo inflamatório da síndrome de Churg- Strauss tende a afetar arteríolas epineurais menores do que as da vasculite sistêmica.^(12)^

Conclui-se que a síndrome de Churg-Strauss frequentemente se faz acompanhar de polineuropatia como complicação; e como a remissão depende de terapia imunossupressora, é importante reconhecê-la na fase inicial. O diagnóstico de polineuropatia baseia-se em estudos clínicos e eletrofisiológicos, mas estudos histológicos, imunoistoquímicos e morfométricos precisos da biópsia do nervo periférico podem ser decisivos para fazer o diagnóstico.

### Poliangeíte granulomatosa

Aproximadamente 50% dos pacientes com poliangeíte granulomatosa apresentam complicações neurológicas causadas por lesões granulomatosas necrotizantes de pequenos vasos.^(1-4,9)^ O comprometimento do SNC inclui várias alterações, dependendo da presença de vasculite, extensão contígua ou propagação granulomatosa remota. Os granulomas causam meningite basilar, disfunção do lobo temporal e oclusão do seio venoso.^(9)^ A neuropatia dos nervos cranianos II, VI e VII é igualmente causada por lesões granulomatosas.^(14)^

A complicação típica e mais frequente do SNP é a mononeurite múltipla (10 a 22%). Também foi relatada polineuropatia sensitivo-motora simétrica com progressão relativamente rápida, embora rara.^(15)^ Com base no Consenso de Chapel Hill, de 1994, o diagnóstico de poliangeíte granulomatosa requer uma biópsia tecidual que apresente evidências de vasculite, demonstradas por inflamação granulomatosa e necrose nos órgãos envolvidos.^(16)^ No tocante ao envolvimento tanto do SNC quanto do SNP, rituximabe e infliximabe surgiram como potenciais opções de tratamento para casos refratários.^(17)^ Trinta e três pacientes com doença ativa foram incluídos em um estudo prospectivo aberto, que avaliou o acréscimo do infliximabe à terapia padrão, com o objetivo de alcançar a remissão dentro de um período de acompanhamento médio de 12 meses.^(18)^ Nenhum benefício foi demonstrado com o uso de agentes antifator de necrose tumoral alfa. As estratégias de tratamento atuais têm efeitos adversos substanciais de curto e de longo prazo, e as recidivas são frequentes; portanto, são necessárias abordagens menos tóxicas e mais efetivas.

## Vasculite de vasos de calibre variável

### Síndrome de Behçet

Esse distúrbio multissistêmico de etiologia desconhecida afeta predominantemente pacientes do sexo masculino, apresentando ulcerações orais e genitais, além de uveíte, com base nos Critérios Internacionais para a Doença de Behçet.^(19)^ A histologia revela vasculite de pequenos vasos e depósito perivascular de células inflamatórias nas meninges.

O envolvimento do SNC na doença de Behçet, geralmente conhecido por síndrome neuro-Behçet (NB), ocorre em 30% dos casos, incluindo a forma aguda e a forma crônica progressiva. A forma aguda da NB é caracterizada por meningoencefalite aguda com lesões focais, apresentando áreas de alta intensidade nas imagens ponderadas em T2 ou FLAIR, na ressonância nuclear magnética.^(20)^ A ciclosporina A frequentemente causa NB aguda. Esta responde à terapia com esteroides e geralmente é autolimitada. Em contrapartida, a NB progressiva crônica é caracterizada por demência intratável lentamente progressiva, ataxia e disartria, com elevação persistente de atividade da interleucina 6 (IL-6) no líquido cefalorraquidiano (LCR; mais de 20pg/mL). O envolvimento do SNP é extremamente raro, embora tenham sido relatados casos isolados de polineuropatia simétrica distal e mononeurite múltipla.^(21)^

A NB progressiva crônica é resistente ao tratamento convencional com esteroides, ciclofosfamida ou azatioprina. Estudos recentes relatam a eficácia do metotrexato, em baixas doses, na NB progressiva crônica.^(22)^ No entanto, o tratamento sintomático é limitado a metilprednisolona intravenosa em altas doses, seguida por administração oral prolongada em doses decrescentes. Por outro lado, o tratamento preventivo inclui azatioprina, ciclofosfamida, interferon alfa e os agentes antifator de necrose tumoral alfa por longo prazo, apesar de não haver evidência em estudos multicêntricos sobre sua eficácia.^(23)^

## Vasculite de órgão único

### Angeíte primária do sistema nervoso central

A angeíte primária do SNC (APSNC) é uma doença inflamatória rara que pode envolver tanto as pequenas quanto as médias artérias leptomeníngeas, corticais e subcorticais do SNC. Os achados histológicos da APSNC são inflamação granulomatosa, necrose fibrinoide da parede dos vasos ou infiltrados celulares exclusivamente linfocitários.^(24-26)^ A forma meníngea subaguda ou crônica é observada principalmente em pacientes do sexo masculino e apresenta-se como cefaleia ou enxaqueca de duração de 3 a 6 meses, seguida de sintomas neurológicos focais e generalizados. Confusão, défice de memória e concentração são sinais de disfunção cognitiva. Outros sintomas do SNC incluem hemiparesia, convulsões, ataxia e paralisia de nervo craniano. O exame do LCR mostra ligeiro aumento de células e albumina. No entanto, os achados clínicos e radiológicos cerebrais são relativamente inespecíficos, de modo que o diagnóstico continua difícil. A biópsia do cérebro revela as lesões histológicas características da doença.^(27)^ A síndrome de vasoconstrição cerebral reversível é a principal afecção que mimetiza a APSNC com cefaleia em trovoada recorrente, com ou sem défice neurológico, e resultados normais dos exames do LCR. A angiografia por ressonância magnética mostra vasoespasmo segmental difuso e reversível dos vasos intracranianos.^(28)^ Como não foi realizado nenhum estudo controlado do tratamento de angeítes do SNC, as recomendações para o tratamento da APSNC se baseiam nos protocolos para vasculites sistêmicas com grave envolvimento de órgãos. Recomenda-se uma combinação de esteroides e pulso de ciclofosfamida para pacientes com mau prognóstico.^(24,25)^ Com índice de recidiva de 25% e de sobrevida muito reduzido, recomenda-se a obrigatoriedade de um acompanhamento minucioso, quando houver suspeita de APSNC.

## Vasculites secundárias a doenças do tecido conjuntivo

### Artrite reumatoide

AR é a doença mais comum do tecido conjuntivo e afeta principalmente as articulações. Os sintomas do sistema nervoso devem-se principalmente à vasculite e às lesões resultantes da pressão causada pela presença de nódulos reumatoides.^(1,5,29)^ A incidência de vasculite reumatoide diminuiu substancialmente nos últimos tempos. O envolvimento do SNC é incomum na AR e apresenta sintomas semelhantes aos da vasculite, como AVC, convulsões e meningite (provavelmente devido aos nódulos reumatoides).

Teorias recentes sobre a patogênese da AR sugerem que as células sinoviais desses pacientes expressam cronicamente um antígeno que desencadeia a produção do fator reumatoide envolvendo o infiltrado de leucócitos polimorfonucleares. A sinovite destrutiva resulta em frouxidão de ligamentos e erosão óssea, sendo a subluxação atlantoaxial a deformidade cervical mais comumente associada à AR. Como são observados défices neurológicos em apenas 7 a 34% dos casos, muitos pacientes com dor e critérios radiográficos para instabilidade não desenvolvem sequelas neurológicas. Porém 10% dos pacientes vão a óbito, devido a compressão do tronco cerebral.^(30)^

As complicações do SNP são muito comuns e dependem da causa, localização e gravidade da lesão: (1) neuropatia sensitiva periférica, que é atribuída à vasculite que caracteriza a doença, embora a patogênese continue desconhecida. De acordo com os achados clínicos e eletrofisiológicos, são relatados prejuízos estéticos em mais de 75% dos pacientes. A neuropatia é predominante axial e se manifesta com sintomas sensitivos leves nas extremidades, caracterizando-se por uma evolução simétrica e progressiva da doença;^(2,3) ^(2) a mononeurite múltipla é menos frequente na AR, mas tem uma evolução clínica mais grave;^(2)^ (3) a neuropatia periférica por compressão é causada por compressão direta de um nervo isolado. Os nervos mais comumente afetados são mediano (síndrome do túnel do carpo); ulnar, na altura do cotovelo; canal de Guyon, na altura do punho; plantar medial ou lateral, no canal do tarso; e fibular;^(2,3)^ (4) a neuropatia periférica sensitivo-motora dos membros superiores e inferiores é mais grave e tem pior prognóstico.^(2,3)^

Fraqueza e atrofia muscular, especialmente em localização proximal, são frequentes em pacientes com AR. A biópsia muscular mostra evidências de miosite em cerca de 40% dos pacientes, sendo que o nível de enzimas musculares está sempre elevado.^(29)^

O tratamento da AR pode causar efeitos colaterais neurológicos. O ouro é a causa da síndrome de Guillain-Barré em 1% dos pacientes, caracterizada pelo aparecimento repentino de fraqueza muscular rapidamente progressiva. Embora raras, foram observadas paralisia de nervos cranianos, mielite transversa e convulsões. A cloroquina pode causar dores de cabeça, perturbações psicóticas, neuropatia e miopatia, ao passo que a D-penicilamina é frequentemente responsável por distúrbios do paladar, miopatia inflamatória ou miastenia. Distúrbios de audição, particularmente a altas frequências, estão associados a doses elevadas de salicilatos, bem como aos efeitos secundários conhecidos dos corticosteroides. A desmielinização observada em pacientes com AR que recebem tratamento com agentes antifator de necrose tumoral alfa pode ser atribuída ao desencadeamento de esclerose múltipla (EM) latente preexistente, ou ao surgimento de um novo episódio desmielinizante (EM ou semelhante à EM), ou ainda à coexistência incidental das duas doenças.^(31)^ No entanto, a morbidade e a mortalidade continuam a ser elevadas, apesar do tratamento agressivo com agentes biológicos ou ciclofosfamida.

### Lúpus eritematoso sistêmico

O LES é uma doença autoimune caracterizada pelo envolvimento de órgãos de vários sistemas, pela heterogeneidade das características clínicas, e pela variedade no grau de severidade, tendo semelhanças com outras doenças autoimunes. Assim, as doenças que mimetizam o lúpus podem se apresentar como doenças similares ao lúpus (por exemplo, com dois ou três critérios) ou como uma doença que satisfaz os critérios de classificação do LES.^(32)^ Diversos locais podem estar envolvidos no sistema nervoso, com frequência nos estágios iniciais, principalmente entre os jovens.^(1,4,9,33)^

As manifestações do SNC variam de 33 a 75% entre os pacientes com LES, mimetizando outras afecções neurológicas:^(33,34)^

*– *Doença não trombótica do SNC em que os anticorpos contra os componentes estruturais do SNC, complexos imunes, vasculites e lesões não vasculares são considerados os principais responsáveis: caracteriza-se mais comumente por psicoses, convulsões, cefaleia (24 a 72%), disfunção cognitiva e coreia;

*– *A doença trombótica do SNC deve-se provavelmente a processos multifatoriais, como aterosclerose acelerada e estado protrombótico, devido a anticorpos antifosfolípides (aPL).^(34)^ Os pacientes com LES e AVC ou ataques isquêmicos transitórios recorrentes devem ser avaliados para fonte valvar de êmbolos (endocardite de Libman-Sacks) e para aPL, porque a terapia anticoagulante se faz necessária nesses pacientes.

As complicações do SNP no LES são raras, surgindo em aproximadamente 10% dos pacientes, devido à vasculite. A neuropatia do trigêmeo pode preceder em vários anos outros sintomas da doença, sendo a polineuropatia sensitiva ou sensitivo-motora simétrica distal a complicação mais comumente observada, subaguda ou crônica, com sintomas leves a moderados. Foram relatados uns poucos casos de polirradiculoneuropatia desmielinizante inflamatória crônica.^(35)^ Geralmente, as miopatias do LES tomam a forma de miopatias inflamatórias quando aparecem na fase aguda da doença ou como efeitos colaterais secundários, como hipocalemia, resultante do tratamento com corticosteroides ou hidroxiloroquina.^(33)^ Devido à ausência de estudos randomizados controlados, a atual abordagem terapêutica ainda é empírica e se baseia na experiência clínica. Os tratamentos com imunoglobulinas intravenosas, micofenolato de mofetila, rituximabe, dexametasona ou metotrexate intratecal necessitam de mais estudos para confirmar sua utilidade.^(34)^

### Esclerose sistêmica

A esclerose sistêmica é caracterizada por microvasculopatia amplamente disseminada e fibrose tecidual difusa, afetando a pele e outros órgãos sistêmicos, particularmente o coração, os pulmões e o trato gastrintestinal. Anteriormente consideradas um evento raro, as complicações neurológicas da esclerose sistêmica estão sendo cada vez mais reconhecidas.^(1,5)^ Numa recente revisão de 180 estudos, o envolvimento do SNC na esclerose sistêmica caracterizava-se por cefaleia (23,73%), convulsões (13,56%) e défice cognitivo (8,47%). Foram frequentemente observadas depressão e ansiedade (73,15% e 23,95%, respectivamente).^(36)^ A neuropatia periférica mais comumente relatada nos pacientes com esclerose sistêmica foi a síndrome do túnel do carpo (1 a 10%).^(2,3,34,36)^ A neuropatia trigeminal ocorreu com mais frequência em pacientes jovens do sexo feminino com esclerose sistêmica, sobrepondo-se a outros distúrbios, principalmente doenças mistas do tecido conjuntivo com evidência clínica de miosite.^(36)^ Alguns pacientes sentem fraqueza muscular proximal e apresentam elevação de enzimas musculares, mas poucos mostram evidência de miopatia inflamatória na biópsia muscular. Os corticosteroides e a ciclofosfamida podem ser eficazes.^(34,36)^

## Síndrome de Sjögren

A SS é caracterizada por infiltrado de células T (CD4+) e destruição das glândulas salivares e lacrimais, levando à perda da produção de lágrimas (ceratoconjuntivite seca) e da saliva (xerostomia). As manifestações neurológicas podem preceder os sintomas da ceratoconjuntivite seca em 40 a 93% dos casos. As complicações do SNC observadas em 15% dos pacientes incluem nevralgia do trigêmeo, AVC, hemorragia, convulsões, meningoencefalite asséptica e mielite transversa;^(34)^ entretanto, o espectro de complicações neurológicas não está bem definido. A lesão vascular pode estar relacionada à presença de anticorpos antineuronais e anticorpos anti-Ro.^(4)^ Além disso, a SS com doença do SNC pode mimetizar a EM, sugerindo outros mecanismos além da vasculopatia.^(1,4,37)^ O SNP é o mais comumente afetado em 30% dos pacientes, principalmente nos mulheres acometidas por SS com polineuropatia sensitivo-motora simétrica periférica e mononeurite múltipla.^(2,3,38)^ Citoquinas pró-inflamatórias, como o fator de necrose tumoral alfa, foram implicadas, e alguma melhora clínica foi observada com terapia de imunoglobulina intravenosa e antifator de necrose tumoral alfa. Observou-se recentemente que anticorpos contra o receptor muscarínico tipo 3 podem explicar parte da disfunção autonômica mais ampla encontrada em pacientes com SS.^(39)^ Embora as complicações do SNC e do SNP na SS sejam difíceis de avaliar, em parte por causa do amplo espectro de manifestações possíveis, sua incidência estimada é de aproximadamente 20%. A mononeuropatia continua a complicação mais específica.

## CONCLUSÃO

As vasculites e as doenças do tecido conjuntivo a elas associadas proporcionam oportunidades para investigar a fisiopatologia do sistema imunológico dos vasos dos sistemas nervosos central e periférico. A prevalência relatada do envolvimento do sistema nervoso central varia muito; no entanto, é geralmente considerada menos frequente do que a do sistema nervoso periférico. A manifestação mais frequente do sistema nervoso periférico é uma neuropatia puramente sensitiva e, em geral, de evolução insidiosa e a longo prazo. As manifestações neurológicas são causadas principalmente por efeito direto da doença, além de efeitos colaterais das imunoterapias. Pesquisando a bibliografia internacional, não foi encontrada nenhuma ferramenta diagnóstica estabelecida nem um tratamento eficaz para esses distúrbios. No entanto, devem ser levadas em consideração, nesta revisão, algumas limitações, como o fato de os métodos de notificação de complicações variarem consideravelmente, e as frequências relatadas terem sido determinadas a partir de estudos com amostras de tamanho variado, envolvendo muitas faixas etárias. Justificam-se estudos clínicos prospectivos para a identificação precoce de todos os possíveis efeitos colaterais dos medicamentos. É importante a avaliação a longo prazo dos pacientes, para tratamento das recidivas.

### Mensagem principal

Nesta revisão, foram atualizadas todas as mais recentes informações referentes a complicações neurológicas, diagnóstico e tratamento, que os médicos devem conhecer.
